# Effect of low energy diet for eight weeks to adults with overweight or obesity on folate, retinol, vitamin B_12_, D and E status and the degree of inflammation: a post hoc analysis of a randomized intervention trial

**DOI:** 10.1186/s12986-018-0263-1

**Published:** 2018-04-10

**Authors:** Nina Rica Wium Geiker, Mette Veller, Louise Kjoelbaek, Jette Jakobsen, Christian Ritz, Anne Raben, Arne Astrup, Janne Kunchel Lorenzen, Lesli H. Larsen, Susanne Bügel

**Affiliations:** 1 Clinical Nutrition Research Unit, Copenhagen University Hospital Herlev-Gentofte, Kildegårdsvej 28, DK-2900 Hellerup, Denmark; 2 Department of Nutrition, Exercise and Sports, University of Copenhagen, Rolighedsvej 30, DK-2000 Frederiksberg, Denmark; 3 National Food Institute, Technical University of Denmark, Kemitorvet, DK-2800 Kgs. Lyngby, Denmark

**Keywords:** Vitamin status, LED, Homocysteine, C-reactive protein, Fat percentage, Vitamin a

## Abstract

**Background:**

Obesity is associated with vitamin insufficiency and low grade inflammation. The purpose of this study was to investigate the effect of weight loss on folate, retinol, vitamin B_12_, D and E status and the degree of inflammation.

**Methods:**

Out of 110, 85 individuals (75% women) aged 39 ± 11 years with a mean ± SD BMI of 33 ± 4 kg/m^2^, completed an eight-week low energy diet (LED). Serum concentration of folate, retinol, B_12_, D and E and C-reactive protein and homocysteine (Hcy) were measured at baseline and at end of the LED.

**Results:**

At baseline, 8% of the participants were deficient in folate, 13% in vitamin B_12_, 2% in retinol, 28% in vitamin D (72% were insufficient in vitamin D), and none were deficient in vitamin E. At baseline, BMI was inversely associated with retinol (*P* < 0.05) as was total and abdominal fat percentage with folate (*P* < 0.05); further BMI and measures of adiposity were positively associated with CRP (*P* < 0.01) and Hcy (*P* < 0.05). Homocysteine was inversely associated with all vitamins but retinol (*P* < 0.001). After the LED, the participants lost a mean [95% confidence intervals] of 12.3 [− 13.1,-11.6] kg. The serum concentration of folate, vitamin B_12_ and D were increased (*P* < 0.001) after the LED whereas the concentration of retinol and vitamin E were reduced (*P* < 0.001).

**Conclusion:**

Eight-weeks LED resulted in 13% weight loss and an increase in the serum concentrations of folate, vitamin B_12_ and D. Baseline adiposity was inversely associated with folate and retinol, and positively associated with markers of inflammation.

**Trial registration:**

Ethical Committee of Copenhagen as no. H-4-2013-135, NCT01561131.

## Background

Observational studies have found negative associations between obesity and the status of vitamins, including folate, vitamin B_12_, A, D and E in multiple ethnicities [1–6]. Given the high prevalence of overweight and obesity, especially in the developed countries, vitamin deficiencies might occur to a greater extent than currently acknowledged [7]. However, little is known about causality for the observed associations between obesity and vitamin status.

In a review from 2009, Garcia and colleagues [6] proposed that either an energy-dense but micronutrient insufficient diet consumed by individuals who are overweight or obese could be the reason for the association or that overweight and obesity induces physiological changes in the metabolism of vitamins. Obesity, is associated with low grade inflammation and these physiological changes have in several studies been shown to be associated with elevated C-reactive protein (CRP) and homocysteine (Hcy) and to low concentrations of retinol among overweight/obese individuals [2, 8–10]. Furthermore, vitamin D is suggested to be sequestered in adipose tissue due to its fat-soluble nature, thereby hindering it from circulatory release and usage, and thus decreasing serum concentrations in obese individuals [11]. Therefore, we hypothesize that the vitamin status for overweight and obese individuals will be lower than in the average Danish population and that weight loss will improve vitamin status.

The aim of this study was to investigate the association between degree of obesity and serum concentration of the vitamins A (retinol), B_9_ (folate), B_12_, D, and E (α-tocopherol) among individuals who are overweight and obese and to investigate the effect of weight loss by low energy diet (LED) on vitamin status and low-grade inflammation.

## Methods

### Study design

The present study is a post hoc analysis of data from an eight-week weight loss period of a study study designed to evaluate subsequent weight maintenance. Data included are based on analyses performed on bio-banked serum samples (Danish Data Protection Agency: 2007–54-0269) from January to July, 2012. The PROKA study was a double-blinded randomized controlled intervention with an initial eight-week weight loss period followed by a four-arm parallel 24-week weight maintenance period [12]. All subjects received written and oral information of the study and signed informed consent upon initiation of the study. The study was approved by the Ethical Committee of Copenhagen as no. H-4-2013-135, registered on ClinicalTrials.gov as no. NCT01561131 and performed at the Department of Nutrition, Exercise and Sports, University of Copenhagen, Denmark.

### Participants

**Fig. 1:**
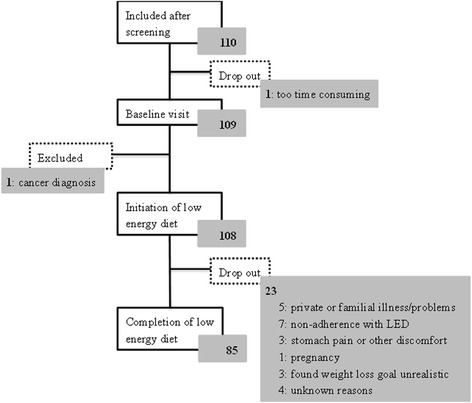
Flow chart. Of the 110 subjects included in the cohort described in the present paper 85 completed the low energy diet

A total of 110 subjects were recruited and 108 initiated the LED (Fig. 1) [12]. Men and women were eligible for the study if they were between 18 to 60 years and had a body mass index (BMI) of 28 to 40 kg/m^2^. Exclusion criterion were: pregnancy (actual or planning hereof in the following 12 months) or lactating, dieting in the past 2 months, weight change above three kilograms prior to study start, surgical obesity treatment, low haemoglobin, smoking, alcohol and drug abuse, inadequate nutrient absorption, chronic endocrine diseases, known chronic systemic infections or inflammation, cardiovascular diseases and cancer in the past 10 years, elite athletes, donation of blood within 3 months of study start or during the study and finally if individuals were evaluated not able to complete an eight-week LED [12]. In total, 85 subjects completed the LED and were included in the present post hoc analysis.

### Diet

During the eight-week intervention (during January–February 2012) participants consumed 3360–4200 kJ per day from LED formula products from Nupo^®^ A/S, Denmark (the used products were approved for total dietary replacement and followed the EFSA guidelines for the composition of these products [13]). The subjects freely chose seven sachets each day of either soups or shakes to dissolve in hot or cold water. In addition, participants were allowed 200 g of water-containing vegetables (tomato, cucumber, radish, celery and lettuce) each day and where prohibited from drinking alcohol. The 8 week weight loss intervention was monitored through five group consultations with a clinical dietician at the site of investigation. The dietician assessed the subjects’ compliance to the LED at each visit.

Prior to baseline participants performed a three-day dietary record where they registered amount (weighed where possible, otherwise household measures were applied), brand names, cooking, and processing of all foods and liquids ingested. Based on these registrations the participants’ intake of energy, macronutrients and vitamins (folate, vitamins B_12_, A, D and E) were calculated using Dankost (DK3000 and Dankost Pro, Copenhagen S, Denmark) including data from Foodcomp (www.foodcomp.dk, National Food Institute, Technical University of Denmark, Søborg, Denmark).

During the LED period the daily intake of vitamin (folate, B_12_, A, D and E), energy and macronutrients was estimated from nutrient labels for seven different products on the webpage of Nupo (18th of June, 2015 at http://www.nupo.dk/da/products/) (Table 2). Since the content of nutrients, apart from vitamins B_12_ and D, varied slightly between products the results are presented as a range of minimum and maximum; the latter including the highest possible energy and nutrient content from the allowed vegetables according to Foodcomp (4th of December, 2015, www.foodcomp.dk, v.7.01, National Food Institute, Technical University of Denmark, Søborg, Denmark).

### Anthropometric data collection

All anthropometric data were collected after an overnight fast and subsequent to recent emptying of the bladder and with the participant only wearing underwear. Body weight was measured to nearest 0.1 kg using a digital scale (Lindells, Malmo, Sweden) and height was calculated as the average of duplicate measures on a wall-mounted stadiometer (Hultafors, Sweden) by nearest 0.5 cm. BMI was calculated as weight (kg) divided by the height squared (m^2^). Waist and hip circumference were the average of two measured to the nearest 0.5 cm with a non-elastic measuring tape. Waist circumference was measured in the midst of the iliac crest and the lowest rib while the participant exhaled and hip circumference was measured at the largest girth in the hip region. With the participants in a recumbent position, the sagittal diameter was measured using an abdominal caliper (Holtain-Kahn, Wales, UK) and fat mass (kg, %) was measured by DXA scan (Lunar iDXA, Madison, USA).

### Blood sampling

Blood samples were collected before and after weight loss after an overnight fast of minimum 12 h by venepuncture of the arm. Samples for folate, retinol, vitamin D, E and CRP were collected in tubes with serum clot activator (Vacuette, Reference No. 454204, Greiner Bio-One, Kremsmünster, Austria) while vitamin B_12_ and Hcy were collected in tubes without additives (Vacuette, Reference No. 456085, Greiner Bio-One, Kremsmünster, Austria). All samples were allowed to coagulate at room temperature for 30 min and then centrifuged at 2500×*g* for 10 min at 4 °C.

### Analytical methods

Serum concentrations of folate, vitamin B_12_ and Hcy were determined using competitive enzyme immunoassay (competitive ELISA). Folate analysis was conducted on IMMULITE® 2000 Systems and vitamin B_12_ and Hcy analysis were conducted concurrently on IMMULITE® 1000 (both from Siemens Medical Solutions Diagnostics, New Jersey, USA). Analyses were conducted using compound-specific commercially available kits (Catalogue No.: L2KFO2 (folate), LKVB1 (vitamin B_12_) and LKHO1 (Hcy); Siemens Healthcare Diagnostics Products Ltd., Llanberis, United Kingdom) and these compounds were determined in singlets. The precision estimated from an internal human serum control varied from 3.1 to 8.2% for folate, vitamin B_12_ and Hcy analyses (not applicable for CRP analysis). Three levels of external control samples were applied for folate, vitamin B_12_, Hcy analysis (Bio-Rad Laboratories, California, USA) and CRP analysis (Horiba ABX SAS, Montpellier, France).

Alfa-tocopherol and all-trans retinol were determined by the method described by Kirkegaard et al. [14]. The precision estimated from doublets of the samples (*n* = 11) analysed on different days were 3.3% and 3.9% for retinol and α-tocopherol, respectively.

The levels of 25-hydroxyvitamin D (25(OH)D) in serum were analysed as described in detail by Burild et al. [15]. The precision estimated from doublets of the samples (*n* = 21) analysed on different days was 5.8%.

CRP serum concentrations were determined in singlets by latex-enhanced immunoturbidimetric assay on ABX Pentra 400 (Horiba ABX, Montpellier, France) using a commercially available kit (Catalogue No.: A11A01611, Horiba ABX, Montpellier, France). The lower limit of quantification for CRP was 0.10 mg/L. A cut-off for CRP ≥190 nmol/L was set based on experience from clinical practice [16] to detect high levels of CRP, unlikely to be caused by obesity-induced inflammation alone; subjects with CRP concentrations ≥190 nmol/L were excluded.

### Data analysis

The following references were used: Low status of serum folate < 6.8 nmol/L and vitamin B_12_ < 148 pmol/L. Insufficiency of serum 25(OH)D < 50 nmol/L and deficiency < 25 nmol/L [17]. The reference interval of retinol 1.05 to 3.90 μmol/L, α-tocopherol 12 to 42 μmol/L, Hcy 5.0 to 12.0 μmol/L and reference for elevated level of CRP < 95 nmol/L.

Measurements of serum concentrations below their respective limits of quantification on apparatuses were set equal to two thirds of the limit value. Additionally, measurements above the limits of quantification were set equal to the limit value (relevant for folate only). Dietary information was obtained from 76 participants.

Baseline data were presented as mean ± SD, if normally distributed, or median (interquartile range). Paired t-tests were applied to check whether baseline data changed significantly after LED. Prevalence before and after weight loss were compared by means of Pearson chi-square tests (for 2-by-2 contingency tables). Associations between serum vitamin concentrations and multiple predictor variables were evaluated for data from before the LED intervention using analysis of covariance (ANCOVA). The vitamin concentrations were transformed using the natural logarithm prior to fitting ANCOVA (if not normally distributed) and the included predictor variables were BMI, waist circumference, sagittal diameter, body fat percentage (total, abdominal and hip region), CRP, and Hcy. Subsequently, regression coefficients and standard errors were “back-transformed” applying the method described by Laursen and colleagues [18]. In case a significant association between an outcome and a predictor was found, the interaction between the predictor and dichotomized CRP and Hcy according to reference levels was evaluated by means of ANCOVA. If an interaction was significant, ANCOVA on subgroups of high and low concentrations was also performed. All ANCOVA models included age and sex as covariates and, additionally month (February to May) of WL initiation when testing serum vitamin D concentrations as outcome. The statistical software R [19] (R Core Team, Vienna, Austria) and Excel (Microsoft, Washington, USA) were used and a significance level of 5% was applied throughout.

## Results

A total of 85 subjects (21 (25%) men and 64 (75%) women) with a mean age of 39 ± 11 years were included in the analyses (Fig. 1). At baseline, the median serum concentration of 25(OH)D was insufficient whilst all other median serum concentrations of other vitamins, CRP and Hcy were within reference values (Table 1).

**Table 1:** Characteristics of the study population (n = 85) before and after eight-week LED and the change with corresponding levels of significance

	Baseline	After LED	Change
*Anthropometry*			
Body weight, kg	95.6 ± 13.9	83.3 ± 12.0	− 12.3 [− 13.1,-11.6]***
Height, m	1.71 ± 0.07		
BMI, kg/m^2^	32.7 ± 3.5	28.5 ± 3.2	− 4.2 [− 4.4,-4.0]***
Waist circumference, cm	102 ± 11	91 ± 9	− 11 [− 12,-10]***
Hip circumference, cm	116 ± 9	108 ± 8	− 8 [− 9,-7]***
Waist-hip ratio	0.89 ± 0.09	0.85 ± 0.8	− 0.04 [− 0.05,-0.03]***
Sagittal diameter, cm ^b^	23.9 ± 2.5	20.1 ± 2.0	− 3.7 [− 4.0,-3.5]***
*Body fat*			
Total, kg	40 ± 9	32 ± 9	− 8.2 [− 8.8,-7.7]***
Total, %	42 ± 6	38 ± 7	− 4.0 [− 4.4,-3.6]***
Abdominal region, %	50 ± 6	44 ± 9	− 6.4 [− 7.4,-5.5]***
Hip region, %	44 ± 7	41 ± 8	− 3.5 [− 3.8,-3.1]***
*Serum concentrations*			
CRP, nmol/L ^a, b,^	22.9 (5.7,41.9)	4.8 (1,33.3)	− 12.4 [− 20,-4.8]*
Hcy, μmol/L	10.5 (8.3,12.7)	9.2 (7.9,11.1)	− 1.4 [− 2.2,-0.7]**
Folate, nmol/L	10.9 (8.6,15.0)	36.9 (26.7,46.9)	23.6 [21.3,26.1]***
Vitamin B12, pmol/L	267 ± 114	332 ± 133	65 [49,80]***
Retinol, μmol/L	1.8 ± 0.4	1.5 ± 0.4	− 0.4 [− 0.4,-0.3]***
25(OH)D, nmol/L	36 (22,55)	50 (41,65)	15 [12,17]***
α-tocopherol, μmol/L	28 (24,33)	21 (18,24)	−7 [−9,-6]***

Results before and after weight loss are presented as mean ± SD or median (interquartile range), changes are presented as mean [95% confidence intervals]; ^a^*n* = 81 at baseline and for change; ^b^*n* = 84 at endpoint; 25(OH)D, 25-hydroxy vitamin D; CRP, C-reactive protein; Hcy, homocysteine; LED, low energy diet; * *P* < 0.01; **P < 0.001; ***P < 0.00000001

The subjects’(*n* = 76) daily energy intake at baseline was 10,238 ± 3247 kJ, and the energy percentage was 17 from protein, 37 from fat and 49 from carbohydrate (Table 2). During the LED period energy intake was reduced by approximately 70% to a mean value of 3259 kJ with protein supplying 50 energy percentage. In their baseline diet subjects met the nutritional recommendations of all vitamins except for vitamin D. The formula diet provided more folate, vitamin A and D than did the subjects’baseline diets but less of vitamins B_12_ and E. The LED did not supply sufficient vitamin D and E to meet the nutritional recommendations (Table 2).

**Table 2:** Mean ± SD of three days dietary records before the intervention (*n* = 76) and the estimated possible range of intake according to the low energy diet and vegetables allowed during the intervention

	Before intervention	During intervention
Energy intake, kJ	10,238 ± 3247	3259–3637
Protein, g	98 ± 32	83–92
Fat, g	101 ± 38	15–20
Carbohydrates, g	295 ± 93	76–102
Alcohol, g	4 ± 12	0
Folate, μg	351 ± 127	932–1098
Vitamin B_12_, μg	6.1 ± 3.0	4.7^a^
Vitamin A, RE	1025 ± 740	1167–1407
Vitamin D, μg	4.7 ± 5.6	8.7^a^
Vitamin E, α-TE	9.6 ± 4.1	7.8–8.4

^a^equal content in all Nupo® sachets. RE: retinol equivalents; α-TE: α-tocopherol equivalents

**Fig. 2:**
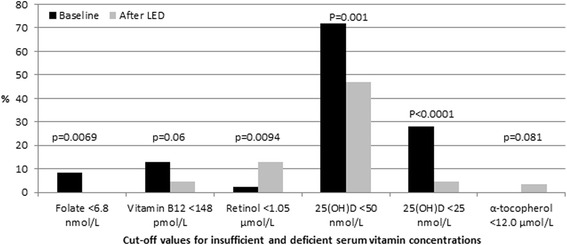
Prevalence of serum vitamin concentrations below reference values before and after weight loss (*n* = 85). 25(OH)D, 25-hydroxyvitamin D

**Fig. 3:**
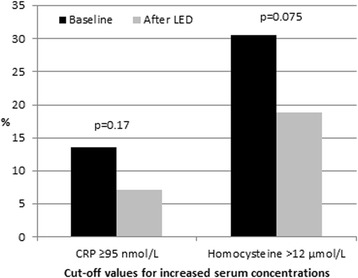
Prevalence of serum concentrations equal to or above reference values for elevated C-reactive protein (CRP) (*n* = 81 at baseline and *n* = 84 at endpoint) and elevated homocysteine (n = 85) before and after weight loss

After 8 weeks LED the subjects lost 13% body weight, 68% of this being fat mass (Table 1). The subjects lost a higher percentage of fat from the abdomen compared to the hip region (13% vs. 8% reduction, respectively). Both CRP and Hcy were reduced after the weight loss, 54% and 13%, respectively. The serum concentrations of folate, vitamin B_12_ and D increased while serum retinol and E (α-tocopherol) were reduced. Fewer subjects were deficient in folate, vitamin B_12_ and D after the LED compared to before the weight loss, while more were deficient in retinol (Fig. 2). According to the reference value of vitamin D where muscle function is found optimal, i.e. 75 nmol/L [20], only six (7%) subjects had 25(OH)D concentration above this threshold at baseline and 15 (18%) after the LED. Additionally, there was a tendency (*P* = 0.075) for fewer participants to have elevated Hcy concentrations after weight loss compared than at baseline; there was no difference in prevalence of elevated CRP (Fig. 3).

### Associations at baseline

At baseline folate and total and abdominal fat percentage were negatively associated as was serum retinol and BMI and waist circumference (Table 3). When stratifying subjects according to CRP (below or above 95 nmol/L), BMI was only negatively associated with retinol (coefficient = − 0.095, *P* = 0.002) among participants with elevated CRP.

**Table 3:** Estimated regression coefficients (slopes) and standard errors (SE) for associations between serum vitamin concentrations and anthropometric predictor variables, C-reactive protein and Hcy (adjusted for sex and age) before weight loss (n = 85)

Predictor variable	Folate	Vitamin B12	Retinol	25(OH)D^a^	α-tocopherol
BMI, kg/m^2^	−0.131 (0.069)	6.72 (4.94)	− 0.035 (0.013)**	− 0.261 (0.247)	− 0.105 (0.209)
Waist, cm	− 0.032 (0.026)	2.54 (1.82)	− 0.012 (0.005)*	− 0.023 (0.092)	0.038 (0.077)
Sagittal diameter, cm ^b^	− 0.115 (0.109)	9.39 (7.76)	− 0.040 (0.021)	− 0.375 (0.404)	0.062 (0.339)
Body fat					
Total body, %	− 0.128 (0.051)*	2.70 (3.76)	− 0.014 (0.010)	− 0.106 (0.187)	− 0.117 (0.157)
Abdomen, %	− 0.107 (0.040)**	− 0.305 (2.93)	0.004 (0.008)	− 0.141 (0.148)	− 0.040 (0.123)
Hip, %	−0.037 (0.037)	1.02 (2.67)	0.002 (0.007)	0.065 (0.132)	−0.112 (0.111)
CRP, nmol/L ^c^	−0.097 (0.065)	1.42 (4.70)	−0.008 (0.013)	0.210 (0.232)	0.306 (0.191)
Hcy, μmol/L	−0.257 (0.045)***	−13.6 (3.38)***	− 0.013 (0.010)	− 0.619 (0.175)***	− 0.383 (0.149)*

Results obtained using analysis of covariance; ^a^additionally adjusted for month of weight loss initiation; ^b^*n* = 81 before weight loss; 25(OH)D, 25-hydroxy vitamin D; CRP, C-reactive protein; Hcy, homocysteine; *P < 0.05; **P < 0.01; ***P < 0.001

At baseline CRP was positively associated with BMI and all measures of adiposity (*P* < 0.01), as was Hcy with BMI, total, and abdominal fat percentage (*P* < 0.05); no associations were observed between CRP and any of the vitamin concentrations. All serum vitamin concentrations (except for retinol) were negatively associated with Hcy concentrations. There were no associations between the changes in serum vitamin concentrations and dichotomized CRP and Hcy.

## Discussion

In the present study we investigated the effect of 8 weeks LED formula products on the vitamin status and low-grade inflammation among overweight and obese individuals. After the 8 weeks the serum concentrations of vitamins A (retinol) and E (α-tocopherol) were reduced, serum concentrations of vitamins B_9_ (folate), B_12_, D were increased and Hcy tended to be reduced. Further, we investigated the association between adiposity grade and serum vitamin concentrations. We observed a high prevalence of vitamin deficiency, particularly in vitamin B_12_ and D among overweight and obese individuals, which is consistent with the literature. And we found BMI at baseline to be inversely associated with serum concentrations of retinol and folate, and positively associated with higher CRP and Hcy. As the used LED products contained vitamins the effect of the 8 weeks LED on vitamin status may be influenced by both weight loss and supplementation; these effects will be included in the following discussion.

### Vitamin concentrations and obesity

As also observed in other studies we found baseline serum concentrations of retinol, folate, B_12_ and D that were lower than in normal weight, but unexpectedly, also lower than observed in other studies of individuals with overweight and obesity [2, 4, 21, 22]. In this study the baseline intake of folate and vitamin B_12_ met the nutritional recommendations, and were equal to or higher than the regular Danish intake [23] but even so, 8–13% of the subjects had insufficient serum concentrations of these two vitamins. Kimmons et al. found an even higher prevalence of folate insufficiency, but have no data on the dietary intake of folate [4]. In a study including 51 postmenopausal women 8 weeks fixed intake of vitamins including folate did not change the association between BMI and the serum concentration of folate [24]. However, in a study of normal weight and morbidly obese subjects Asheim et al. did not find any association with BMI; however, they did not present more precise measures of adiposity [2]. The above results may indicate that increased fat mass causes an imbalance in the metabolism of folate, and due to this, BMI is not an optimal measure. Like us, others find folate associated with other measures of adiposity, i.e. total and abdominal fat percentage [4, 24].

At baseline 70% of the subjects were insufficient in vitamin D and almost 30% were deficient; this is considerably higher than found among a comparable, obese as well as non-obese, population living at the same latitude [25]. Even though the baseline dietary intake of vitamin D was below the recommendation it was double that otherwise found in comparable groups [17, 23]. Compared to non-obese the participants in the present study had a high percentage of body fat that could result in vitamin D being sequestered in the adipose tissue, as it is well recognized to be [11]. When comparing groups of normal weight and obese other studies have found an association between serum vitamin D and obesity [2, 4, 22]. In the present study all the subjects were within the same BMI category and we did not find an association between BMI and vitamin D.

The higher prevalence of deficiency of folate, vitamin B_12_ and D among overweight and obese individuals, as found in our study as well as in other studies, may indicate a higher requirement to attain optimal serum levels to ensure bodily functions, and it may therefore be appropriate to differentiate the recommended intake of at least folate, vitamin B_12_ and D to accommodate this. In the present study the serum concentration of both vitamin D and folate increased significantly after intake of the LED products; these products supplied a higher dosage than did the baseline diet. However, as a fat soluble vitamin, the increase in vitamin D may likely be released from adipose tissue; though not adequately to meet need due to the preexisting deficiency [26], and additional vitamin D either from diet or through supplements is therefore required. Even though the content of vitamin D in the LED products was higher than in the baseline diet it was still below recommendations and the content may have been inadequate to increase the status at January–February were the study was conducted. A previous study by Bügel and colleagues found 10 μg vitamin D supplementation initiated in September, i.e. before the seasonal reduction, to ascertain the serum concentration during winter [27]. For individuals with obesity and suboptimal vitamin status the results from this study indicate the content of all vitamins, but folic acid, should be higher than in the used LED products to reach optimal status.

In the present study, the baseline serum concentration of retinol was similar to what was found in obese Norwegians [2]. But compared to normal weight, overweight and obese American nurses, we found a higher prevalence of retinol deficiency [4]; this could be explained by a high intake of the vitamin A fortified food products available in the US which are not offered in Denmark.

In accordance with findings in other studies among overweight and obese men and women, we found a negative correlation between baseline serum retinol concentration and BMI and waist circumference [28, 29]. The principal carrier of vitamin A (in the form of retinol) is retinol binding protein 4 (RBP-4), which predominantly is secreted by the liver and adipocytes [30, 31]. During the LED the supplied amount of vitamin A was equivalent to the subjects’ baseline intake and within the recommended intake [17], but even so the serum concentration of retinol decreased during weight loss. Vitamin A is fat soluble and therefore requires fat for absorption. The LED products were low in fat, but every meal supplied subjects with a small amount of fat in combination with the vitamins. As the vitamin A in the LED was given as retinol the low fat content in the diet should not have any effect on the absorption [32, 33], and it is therefore not likely to be the cause of the observed reduction in serum retinol concentration. Other studies have found both dietary and surgically induced weight loss to reduce the RBP-4 expression and, among obese, also circulating RBP-4 concentration [31, 34, 35]. In the current study we observed a marked reduction in adipose tissue (i.e. adipocyte shrinkage) with a 10.5% simultaneously increase in prevalence of retinol deficiency. Though we did not analyse RBP-4 in the present study, the observed results indicate a reduction in transporting proteins caused by weight loss. Since the LED provided an equal amount of vitamin A the reduction in serum retinol concentration during this period is more likely to be decreased levels of RBP-4 than it is a reflection of a lower vitamin A status.

The vitamins in the LED products were synthetic which may have reduced the bioavailability of some, e.g. retinol, and increased it for others, e.g. folic acid. Other dietary factors such as animal-to-plant ratio are also found to influence the bioavailability [36], thus individual differences in the LED products could affect the overall bioavailability of the added vitamins. We did not register the individual subjects’ formula product composition thus the effect of other dietary factors on vitamin bioavailability is merely speculative.

Just over 30% of the subjects had elevated levels of Hcy, indicating mild hyperhomocysteinemia which may occur due to insufficient remethylation caused by lack of folate and vitamin B_12_ [37]. Not only did we find 8–13% of the subjects insufficient in these two vitamins, we also found an inverse relationship between Hcy and folate and vitamin B_12_ as well as with measures of adiposity. After the LED, the serum concentration of folate and vitamin B_12_ increased significantly and we simultaneously found a reduction of serum Hcy concentration. Several studies have previously found reduction of Hcy in response to increased intake of folate and vitamin B_12_ [37]. As the LED supplied less vitamin B_12_ than did the subjects’baseline diet, the increase in the serum concentration of vitamin B_12_ may indicate a higher bioavailability or other positive circumstances during the LED period.

### Limitations

We relied on self-reported information to assess dietary intake from the obese individuals. This poses a risk of underestimation; in particular of energy dense foods with low micronutrient content. Furthermore the recording period of 3 days may have caused a potential risk of underreporting rarely consumed foods. The subjects included in the present study were volunteers and therefore anticipated to be motivated for correct registration of their dietary intake. To support this, we found a higher energy intake and slightly lower micronutrient density than found in the general Danish population [38]. We did neither exclude participants taking micronutrient supplementation prior to the study or collect information on this. The fact that we only found an inverse association between BMI and serum vitamin D concentration may be due to use of supplements, but since a relatively high proportion of the subjects were insufficient in one or more vitamins a general use of supplements does not seem plausible. Even so, it is a limitation that there is not collected information on use of supplements or specific dietary behaviour such as vegetarian diet. During the LED there was a high adherence to the formula products, which is reflected by the reduction in weight similar to that found in other LED interventions (e.g. [39]).

## Conclusion

Among 85 subjects with obesity eight-week LED resulted in 13% weight loss, whereof 68% was adipose tissue. After the LED the serum concentration of folate, vitamin B12 and D was increased and retinol and vitamin E was decreased. With the setup of the present study it is however not possible to differentiate between the effect of potential recycling of specifically vitamin E and D caused by metabolism of adipose tissue and the vitamin supplementation from the LED.

## Abbreviations

25(OH)D: 25-hydroxyvitamin D
ANCOVA: Analysis of covariance
BMI: Body mass index
Competitive ELISA: Competitive enzyme immunoassay
CRP: C-reactive protein
Hcy: Homocysteine
LED: Low energy diet
RBP-4: Retinol binding protein 4
